# Research on the Application of MEMS Intelligent Sensor in Abnormal Monitoring of Metro Tunnel by Simplified Model Tests

**DOI:** 10.3390/mi13081242

**Published:** 2022-08-02

**Authors:** Yan Gao, Ketian Sun, Jiayi Tian, Xiaodong Wu

**Affiliations:** 1 School of Earth Sciences and Engineering, Sun Yat-sen University, Zhuhai 519082, China; sunkt@mail2.sysu.edu.cn (K.S.); tianjy25@mail2.sysu.edu.cn (J.T.); wuxd6@mail2.sysu.edu.cn (X.W.); 2 Southern Marine Science and Engineering Guangdong Laboratory (Zhuhai), Zhuhai 519082, China

**Keywords:** metro tunnel, MEMS sensor, intelligent monitoring, model test

## Abstract

The current monitoring methods for tunnel structure deformation mainly focus on laser distance measurement, fiber Bragg grating, photogrammetry, electronic total station, hydrostatic leveling and so on. Compared with traditional monitoring methods, MEMS sensors have the advantages of small size, low cost, low energy consumption and high accuracy. In this paper, MEMS sensors are used for the continuous real-time intelligent monitoring of model tunnels, and the multi-point deployment of MEMS sensors is set up for the tunnel structure monitoring with the indicators of acceleration and inclination. The results demonstrated that β-sample interpolation of the angles of the MEMS measurement points, and then integration of the overall displacements can better reflect the form of uneven settlement of the tunnel. For tunnel models with uneven settlement as the main deformation, the angle interpolation method allows the MEMS sensor to measure the vertical displacement more accurately and to determine the load mode to a certain extent. However, for tunnel models with global settlement as the main deformation, the results vary considerably from reality, as only the uneven part of the settlement can be measured using the angular interpolation method.

## 1. Introduction

As society develops by leaps and bounds, ground space alone can no longer meet the traffic needs of medium and large cities, and the demand for subway construction is increasing day by day. By the end of 2021, 50 cities in mainland China had opened 283 urban rail transit operating lines with a total length of 9206.8 km [1], of which 7209.7 km of metro operating lines, reaching 78.3% of the total length, were the most dominant part. The traditional monitoring methods of subway tunnel deformation have limitations, e.g., a limited number of monitoring points, the inability to use remote measurement, poor performance due to poor visibility and narrow space in the subway tunnel etc. For these reasons, in recent years, some new monitoring equipment, e.g., high-speed laser scanning sensors, mobile detection vehicles, and Electronic Total Stations have been used for high-precision and automatic monitoring in the subway tunnel. Some emerging measurement technologies such as fiber Bragg grating sensing technology and three-dimensional laser scanning have been applied to deformation monitoring. Although the above technical means have developed relatively, there are still obvious application limitations, such as high production cost of measuring instruments, limited measurement range, limited detection points, ease of damage, and extreme sensitivity to environmental factors such as variable temperature, etc. [2,3,4,5,6,7,8]. Therefore, there is still a need for continuous innovation and improvement in tunnel deformation monitoring, and the development of low-cost, high-precision, intelligent monitoring tools that can be used over a large area.

Micro-electro-mechanical system (MEMS) sensors are the product of mechanical miniaturization machining technology. With the development of microelectronic circuit technology and chip processing technology, MEMS sensors are suitable for large-scale application in industry and engineering due to their low cost, small size, light weight, low power consumption, high reliability, high sensitivity, easy integration, and suitability for harsh working environments.

The origin of MEMSs can be traced back to the 1950s. In 1962, the advent of the first silicon micro pressure sensor pioneered MEMS technology, and the progress and development of MEMS technology contributed to the improvement of sensor performance [9].

In 1987, Feng et al. developed a movable silicon miniature electrostatic motor, which meant that scientists turned from sensor research to MEMS research and attracted much attention from countries all over the world, and set off a climax of technological innovation in the MEMS field [10]. With the advantages of small size, light weight, low power consumption, high reliability, high sensitivity, easy integration and suitability for harsh working environments, MEMS sensors are developing rapidly, and various countries are taking MEMS sensor technology as one of their strategic directions. Bennett et al. [11,12] used wireless sensor network (WSN) technology based on MEMSs to monitor the subway tunnels in London and Shanghai, which demonstrated the feasibility and advantages of wireless sensing in the underground environment, but due to the complex movement of the target structure, a deviation between the results of each sensor was developed. Through WSN technology based on MEMSs, Yu [13] explored the application of wireless sensing technology in the subway shield tunnel and studied the influences of measuring the point arrangement of WSN and different shield construction parameters on adjacent areas. Zhang [14] put forward an underground engineering monitoring method based on the wireless inclination sensor. However, the final monitoring data were not continuous due to the unavailability of a fixed power supply required for the continuous operation of the base station of the wireless monitoring system during the field test. Zhang et al. [15] established a tunnel health monitoring system based on WSN and developed and assembled wireless sensors for the internal and surface health monitoring of tunnel structures. However, partial data loss was found during the actual test in the London underground tunnel.

Although MEMS sensors have been used in more applications in China and abroad, they are currently used in geological engineering and civil engineering mainly for bridge and slope monitoring [16,17,18,19,20,21,22,23] and are still lacking in tunneling [16,24,25,26,27]. Based on model experiments, the feasibility and precision of applying MEMS sensors in tunnel engineering are explored. By simulating different deformation forms of a tunnel under various working conditions, including single-tunnel and cross-tunnel, lining simulations with various strengths and wall thicknesses, the law of response and monitoring accuracy of MEMS sensors to prolonged periods of deformation can be obtained.

## 2. Experimental Design of Tunnel Anomaly Monitoring Based on MEMS

### 2.1. Test Materials and Grouping

In the tests, natural river sand was used as the overburden layer of the tunnel, with the particle size mainly ranging from 0.25 to 1 mm, a natural moisture content of 10%, a natural density of 1500 kg/m^3^, an initial pore ratio of 0.69 and internal friction angle of 30.

Since most metro tunnels have a shape similar to a long straight cylinder, in the model test during this study, the metro tunnel was simplified and mainly simulated by a long straight cylindrical tube. The complex structure was not considered. Different materials were used to simulate the lining strength of different tunnels, i.e., acrylic and 304 stainless steel are commonly used to simulate tunnel materials [28,29,30]. Acrylic tubes were used to characterize the weaker tunnel lining strength while 304 stainless steel tubes were used to characterize the stronger tunnel lining strength to investigate the deformation response of different tunnels.

**Figure 1:**
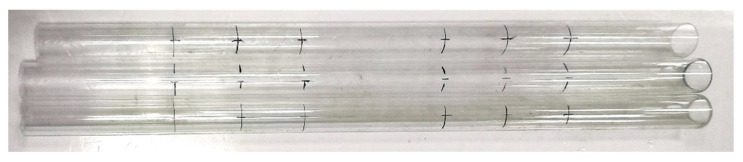
Acrylic pipes used in the test. The wall thickness is 1 mm, 2 mm, and 3 mm from top to bottom respectively.

**Figure 2:**
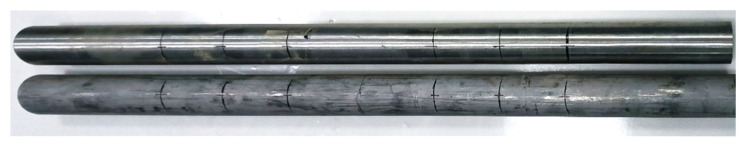
The 304 stainless steel pipes used in the test. The wall thickness is 0.1 mm and 0.2 mm from top to bottom respectively.

The acrylic tubes used in the tests had an external diameter of 5 cm and internal diameters of 1 mm, 2 mm and 3 mm, as shown in Figure 1. The 304 stainless steel tubes also had an external diameter of 5 cm and wall thicknesses of 0.1 mm and 0.2 mm, respectively, as shown in Figure 2. The specific experimental groups are shown in Table 1.

The Materials Test Systems with a maximum force rating of 100 kN (MTS) were used to deploy pressurization. Concentrated loads were applied using a rigid joint of the MTS testing machine with a diameter of 5 cm, which is small in relation to the model size and can be considered a concentrated load. To avoid punching failure of the tunnel model subjected to the concentrated load, the ultimate load tested by the experiment was 220 N, hence, 200 N was adapted as the testing load and the loading location is the center of the tunnel.

### 2.2. Tunnel Simulation Scheme

The test model box is a 600 × 400 × 400 mm polypropylene thickening model box. Two layout schemes were adopted to simulate authentic environments: a single tunnel and two orthogonal tunnels.

The MEMS sensors’ model is JY901S, which is an integration of a high-precision gyroscope, accelerometer and geomagnetic field module. Through the high-performance microprocessor, advanced dynamic solution and Kalman dynamic filtering algorithm, the current real-time motion of the module can be solved quickly, the measurement noise can be effectively reduced, and the measurement accuracy can be improved. The specifications of the MEMS sensor are shown in Table 2.

#### 2.2.1. Single Tunnel

**Figure 3:**
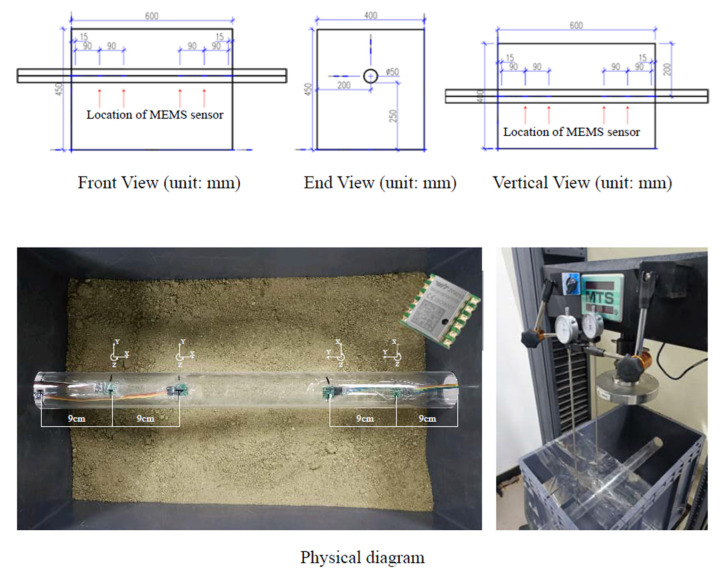
Single tunnel layout scheme. Noted that the scales are not the same.

The tunnel passed through one end of the model box and exited from the other. The specific layout location and size are shown in Figure 3. The four MEMS sensors were arranged at the distance of 9 cm and 18 cm at both ends of the model and fixed on the inside of the tunnel model to avoid the influence of soil and external loads on the sensor. 

All sensors were affixed to the top. Four dial gauges were fixed with external magnetic brackets, located at the top of the MEMS sensor. The thin steel pipe was used to extend the dial gauges to contact the tunnel model directly. An auxiliary structure was arranged above the soil to limit the lateral movement of the thin steel pipe.

#### 2.2.2. Orthogonal Tunnels

**Figure 4:**
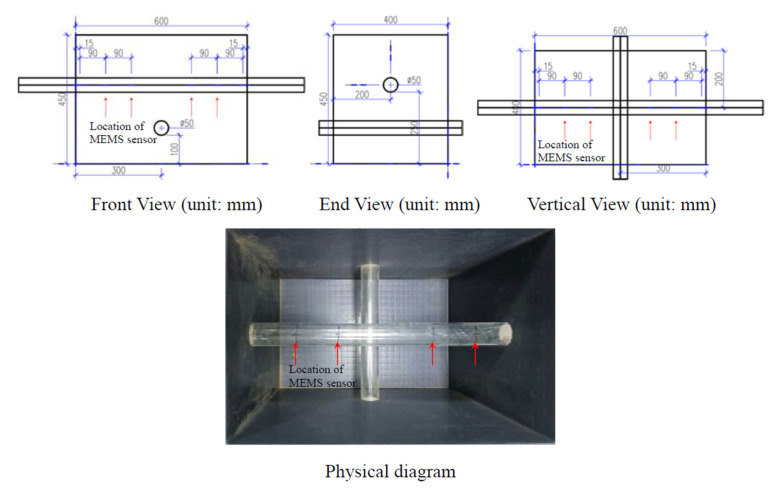
Orthogonal tunnels layout scheme.

The orthogonal tunnels and sensor positions (pointed out as red arrows) were stetted as in Figure 4. Another tube was set under the original signal tube. The locations of the MEMS sensors were the same as the signal tunnel.

## 3. MEMS Data Processing Method

The MEMS sensor method is used to measure settlement as follows: first, a specific time point is determined as the interception point (in all tests, the moment after loading is completed and before the stress relief occurs when the angle of inclination is at its maximum), and the XYZ angle is extracted from the corresponding time of the raw data of the four sensors, and then the true dip of the sensor is calculated by the following geometric operation. The XYZ coordinates for the Euler angles are shown in Figure 3 in the single-tunnel layout scheme. It is noted that the scales are not the same. The rotation order of the Euler angle is defined as Z-Y-X. The sensor returns the Euler angle data because there is no rotation around the Z-axis in the test, so only the rotation angle $\beta_{1}$ around the X-axis and the rotation angle $\beta_{2}$ around the Y-axis are considered. A mathematic relationship can be derived from geometric relations:

(1) $$\tan\beta_{1}=\tan\alpha \times \cos\omega$$

where α is the true dip and ω is the angle between the true dip and the Z-axis. ω is unknown, but another relationship is known:

(2) $$\tan\beta_{2}=\tan\alpha \times \cos\left(\frac{\pi }{2}-\omega \right)$$

From Formulas (1) and (2), a new equation can be obtained

(3) $$\alpha =\arctan\left(\sqrt{\tan^{2}\beta_{1}+\tan^{2}\beta_{2}}\right)$$

The analytical solution of true dip can be calculated by Equation (3). The inclination angles of the four sensors can be extracted by this method, corresponding to their respective installation positions.

The part of the model pipe that is inside the test chamber is then differenced, with the differential cell size set to 1 mm, to obtain a large number of points where the dip values are vacant. Using the β-sample interpolation method to interpolate the inclination angle at known points, the inclination angle within the same differential unit is approximated by the inclination angle of the differential point on the left side, so that the inclination angle values of all differential unit points are obtained, and then the settlement on both sides of each differential unit can be calculated. By stipulating that one end of the tunnel is the 0-settlement point and by accumulating the settlement of each differential unit, the uneven sedimentation of all positions of the model pipeline can be obtained and drawn in the Cartesian coordinate system with the midpoint of the tunnel as the origin, the distance from the origin as the X-axis and the settlement as the Y-axis. Finally, the settlement measured by the dial gauge is added to the above Cartesian coordinate system, which is the final data processing result.

## 4. Deformation Characteristics of the Tunnel under Concentrated Load

### 4.1. Acrylic Pipe Simulated Tunnel

**Figure 5:**
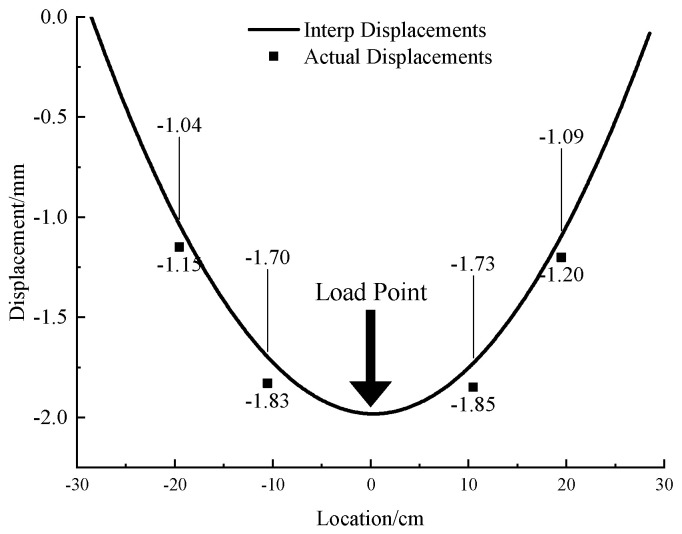
Deformation curve and measured displacement of single acrylic tunnel with 1 mm thickness under concentrated load.

In the 1 mm acrylic pipe test, after data processing, the tunnel displacement measured by the MEMS sensor under the concentrated load is shown in Figure 5. To avoid the influence of boundary conditions, a gap of 1.5 cm was reserved from the boundary as shown in Figure 3 and Figure 4. Hence, the location points of the MEMS sensors in the x-axis in Figure 5 are −19.5 cm, −10.5 cm, 10.5 cm and 19.5 cm. The errors between the interpolation displacement of MEMSs data and the measured displacement at different points are small (absolute error 0.1 mm, relative error 6–10%). Under the concentrated loading, the deformation of the tunnel is symmetrically distributed about the pressure point of the concentrated load, the whole tunnel produces uneven settlement and the horizontal influence range of the uneven settlement is about 12 times the diameter of the tunnel.

**Figure 6:**
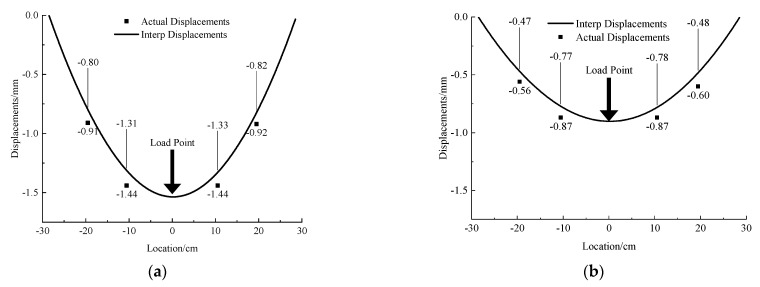
Deformation curve and measured displacement of single acrylic tunnel with 2 mm and 3 mm thickness under concentrated load. (**a**) 2 mm. (**b**) 3 mm.

**Figure 7:**
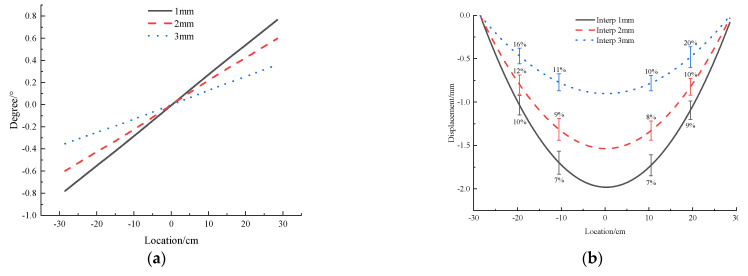
Angle and Displacement Curve of acrylic tunnel with 1 mm, 2 mm and 3 mm thickness under concentrated load. (**a**) Angle. (**b**) Displacement.

The results of 2 mm and 3 mm thickness acrylic pipe are shown in Figure 6, and its deformation pattern is consistent with that of 1 mm thickness acrylic pipe. Comparing the three groups of test results of 1 mm, 2 mm and 3 mm thickness (Figure 7), it can be found that with the increase in the wall thickness of the acrylic model tube, the local rotation angle and displacement of the acrylic model tube decrease gradually under the same external load, and the relative error of the sensor measurement is also gradually increasing. However, the absolute error is relatively stable (about 0.1 mm), which is inferred that the tunnel and soil are compressed under external load, leading to the overall subsidence of the tunnel model. With the increase in the uneven settlement of the tunnel itself, the error caused by the overall settlement decreases.

Therefore, although the current MEMS sensor itself cannot correct the settlement errors caused by the overall settlement of the tunnel, the variation trend of uneven settlement reflected by it is almost the same as that of the measured settlement. When the uneven settlement of the tunnel is large, it can be considered that the interpolation displacement of MEMS data is close to the measured displacement.

### 4.2. 304 Steel Pipe Simulated Tunnel

**Figure 8:**
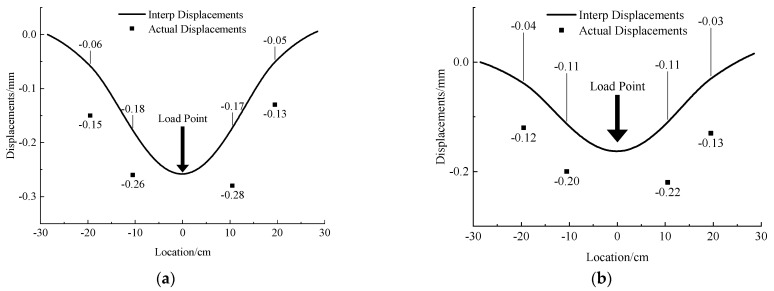
Deformation curve and measured displacement of single steel tunnel with 0.1 mm and 0.2 mm thickness under concentrated load. (**a**) 0.1 mm. (**b**) 0.2 mm.

Under the same external load, the 304 stainless steel was less displaced due to its greater stiffness, with overall displacements in the 0.25 mm range. The deformation curves of the 0.1 mm and 0.2 mm steel pipe simulation tunnels are shown in Figure 8.

For the steel pipe with 0.1 mm wall thickness, under the same external load as the acrylic pipe, the uneven settlement is only 0.1 mm. Therefore, the displacement caused by the compression deformation of the soil layer accounts for the majority of the settlement. In this case, the relative error between the data obtained by the MEMS sensor and the measured data is more than 30%, and even reached 60% at one time, so it is difficult for the sensor to reflect the displacement accurately, but the tunnel deformation can still be qualitatively characterized, and the deformation trend is consistent with the measured curve.

As for the 0.2 mm thickness steel pipe, the test result is the same as that of the 0.1 mm thickness steel pipe. Under greater stiffness, the uneven settlement of the tunnel model is almost zero, and the settlement is dominated by the overall settlement caused by the deformation of the model box. The MEMS sensor can qualitatively reflect the tunnel deformation, but there is a large gap in quantity because the relative error is more than 50% at each measuring point, which means that the measurement is inaccurate.

Therefore, the method of using MEMS sensor interpolation to obtain displacement is not suitable for measuring the deformation dominated by the overall settlement and can only qualitatively characterize the uneven deformation in the whole settlement.

**Figure 9:**
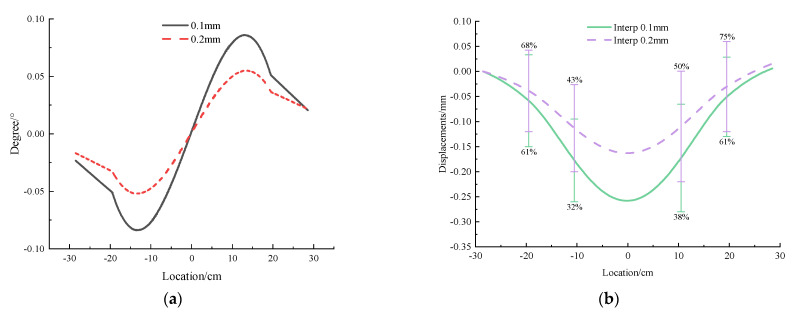
Angle and displacement curve of steel tunnel with 0.1 mm and 0.2 mm thickness under concentrated load. (**a**) Angle. (**b**) Displacement.

Comparing the test results of different wall thicknesses, as shown in Figure 9, it can be found that the deformation decreases with the increase in wall thickness. At the same time, because the angle curve tends to be 0 but does not contact with 0 at both ends in addition to the middle point, it can be considered that the deformation curve is similar to the normal distribution curve. Compared to the acrylic model, the deformation of the steel pipe is smaller, and the shape of the deformation curve is not similar.

## 5. Deformation Characteristics of Orthogonal Tunnels under Concentrated Load

**Figure 10:**
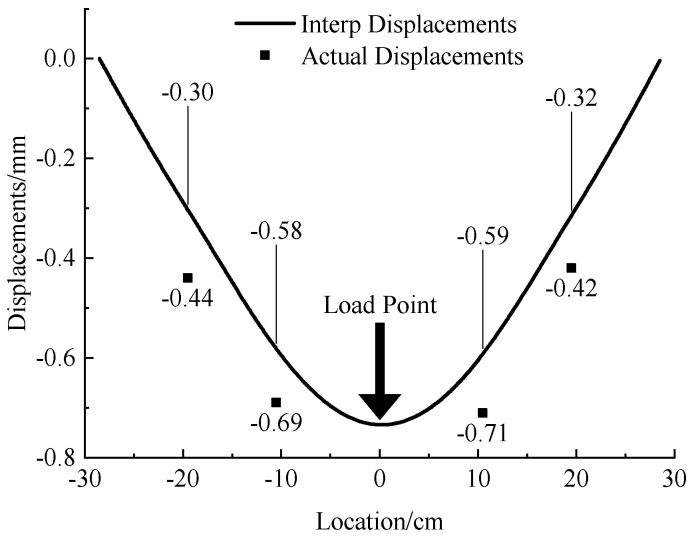
Deformation curve and measured displacement of orthogonal acrylic tunnel with 1 mm+1 mm thickness under concentrated load.

**Figure 11:**
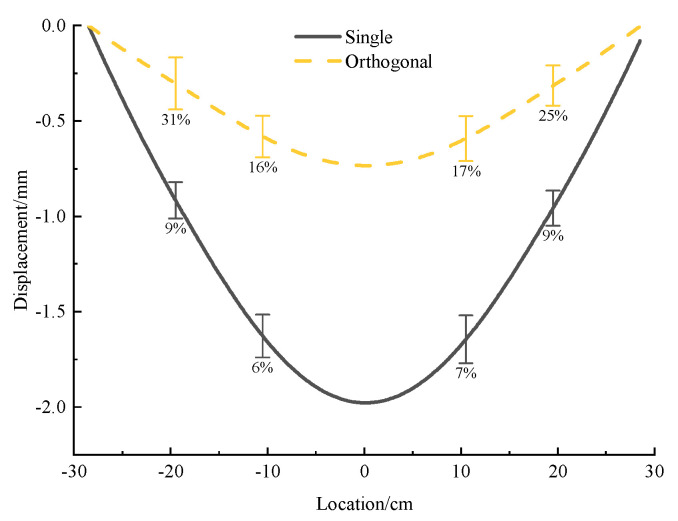
Comparison of deformation between 1 mm single tunnel and 1 mm + 1 mm orthogonal tunnel under concentrated load.

After putting another tunnel below the original single tube as shown in Figure 4, the deformation of the upper tunnel is significantly reduced from the original 1~1.5 mm to 0.4~0.7 mm (a bit more than 50%), as illustrated in Figure 10 and Figure 11, and the deformation curve is transformed towards a V-shape, being close to a straight line on the edge area. The possible reason for the decreasing deformation is that the tunnel below is equivalent to a reinforcement of the whole sand layer for the upper original tunnel, enhancing the strength of the original tunnel instead.

The deformation variation caused by passing through the tunnel is much clearer to be seen on the deformation diagram of the sensor data in Figure 11. The change is bigger in the center area where is near passing through the tunnel, while the change is smaller in the edge area.

## 6. Conclusions

This paper explores the method and accuracy of the MEMS sensor monitoring of tunnel deformation based on MEMS sensors through tunnel model tests, and the main conclusions are as follows.

(1) By studying the principle of MEMS sensors, the study identifies possible detection indicators, including triaxial acceleration and triaxial orientation angle, which were identified and verified through laboratory tests, proving that both types of indicators can reflect the different deformations of the tunnel to a certain extent.

(2) Using the MEMS accelerometer to obtain acceleration and directly obtain quadratic integration to obtain displacement is only feasible for a short period of time. Because the acceleration errors of sensors will be accumulated in the integral operation, the accuracy is reduced over a long-time measurement.

(3) A method is put forward to obtain the non-uniform sedimentation settlement of the whole tunnel by using the inclination angles in measuring points. That is, the tilt angles obtained by MEMS sensors are interpolated by the β-spline to obtain all tilt angles in the whole tunnel; then, the sedimentation settlement difference for each tilt element is obtained by geometric operation, followed by the geometric integration to obtain the whole deformation. With this method, the differential sedimentation settlement of the tunnel can be obtained accurately. When the overall settlement of the tunnel is significant, the overall displacement will also need to be corrected using other methods.

(4) When the overlying loads are the same, the tunnel deformation decreases as the tunnel strength increases. The lower the strength of the tunnel, the greater the uneven settlement caused by the concentrated load, and the higher the accuracy of the displacement obtained using MEMS monitoring of the inclination angle. Tunnel deformation is also related to the way that the tunnel is laid out.

## Figures and Tables

**Table 1:** Grouping design table of test scheme.

Soil Condition	Load Distribution	Tunnel Form	Tunnel Material	Wall Thickness Grouping
Homogeneous soil layer	Concentrated load	Single tunnel	Acrylic	3 mm
				2 mm
				1 mm
			Steel	0.2 mm
				0.1 mm
		Cross orthogonal tunnels	Acrylic	1 mm

**Table 2:** The specifications of the JY901s MEMS sensor.

Parameters	Measuring Range	Resolution
Acceleration	+16 g	0.0005 (g/LSB)
Angular velocity	±2000°/s	0.061 (°/s)/(LSB)
Magnetic field intensity	±2 Gauss	0.0667mGauss/LSB
Pitch, roll angle	X: ±180°, Y: ±90°	0.1° (Static)/0.5° (Dynamic)
Heading angle	Z: ±180°	1°
